# Effectiveness of a positive deviance approach to improve mother’s nutritional knowledge, attitude, self-efficacy, and child’s nutritional status in Maji District, West Omo Zone, South West region, Ethiopia: a cluster randomized control trial

**DOI:** 10.3389/fpubh.2023.1277471

**Published:** 2023-11-13

**Authors:** Abraham Tamirat Gizaw, Pradeep Sopory, Morankar Sudhakar

**Affiliations:** ^1^Department of Health, Behavior, and Society, Institute of Health, Faculty of Public Health, Jimma University, Jimma, Ethiopia; ^2^Department of Communication, Wayne State University, Detroit, MI, United States

**Keywords:** breastfeeding, complementary feeding, CRCT, IYCF, PDA, West Omo Zone, rural Ethiopia

## Abstract

**Background:**

Achieving appropriate feeding for infants and young children continues to be a struggle. These impediments are not only due to limited food availability but also inadequate knowledge, unfavorable attitudes, and low self-efficacy. A positive deviant approach (PDA) addressing positive and possible solutions inherent in a community focusing on problems is applied in Africa and particularly to Ethiopia. Therefore, this trial is aimed at evaluating the effectiveness of PDA in improving mothers’ nutritional knowledge, attitudes, self-efficacy, and children’s nutritional status.

**Method:**

This was a cluster randomized control trial in which 516 mothers were randomly assigned to either an intervention or control group after collecting baseline data. The trial participants in the intervention cluster received a positive deviant intervention for 6 months, whereas those in the control group received only the usual care. Trained positive deviant mothers (PDM) delivered the intervention. A pretested, structured, interviewer-administered questionnaire was used for data collection. Generalized estimating equation regression analysis adjusted for baseline covariates and clustering was used to test the intervention effect.

**Result:**

The results showed that PDA improved breastfeeding outcomes in the intervention groups compared to their counterparts. A mean difference (MD) of breastfeeding (BF) knowledge (MD = 6.47; 95% CI: 6.45–6.49), BF attitude (MD = 12.68; 95% CI: 11.96–13.40), and BF self-efficacy (MD = 3.13; 95% CI: 3.05–3.21) was observed favoring the intervention. The intervention group showed better improvement in complementary feeding (*CF*) knowledge, attitude, and self-efficacy among mothers compared to the control group. A mean difference in *CF* knowledge (MD = 4.53, 95% CI: 4.31–4.75), *CF* attitude (MD = 9.14, 95% CI: 8.52–9.75), and *CF* self-efficacy (MD = 11.64, 95% CI: 11.16–12.12) were observed favoring the intervention. At the end of the 6-month follow-up, children in the intervention group showed a lower prevalence of underweight (18.23%) (95% CI: 4.55, 22.54%; *p* = 0.004) compared with the control group.

**Conclusion:**

PDA was effective in improving mothers’ nutritional knowledge, attitude, and self-efficacy and reducing children’s underweight in the intervention area.

**Clinical trial registration:**ClinicalTrials.gov, identifier PACTR202108880303760.

## Introduction

Around the world, a total of 156 million children experienced stunted growth, and 50 million were wasted. It has been found that only about 43% of infants are exclusively breastfed, while the majority of children do not receive sufficient and safe complementary foods. Additionally, in numerous countries, less than a quarter of infants aged 6–24 months meet the required standards for dietary diversity and feeding frequency that are appropriate for their age (1, 2). Ethiopia is making efforts to implement a comprehensive nutrition plan but faces challenges in promoting optimal infant and young child feeding practices. A national survey revealed that almost half of infants under 6 months aren’t exclusively breastfed, one in four receive pre-lacteal feeds, and only half are breastfed within an hour after birth. Additionally, less than 10% of children under 24 months achieve minimum dietary diversity, and only 6% meet the criteria for a minimum acceptable diet (3). The growth of children is influenced by multiple factors, such as their economic status, childrearing practices, health, and nutritional status. Eating behavior directly affects nutritional status and development (4).

The Positive Deviance Approach (PDA) in the field of health refers to the phenomenon where certain individuals achieve positive outcomes despite challenging circumstances. In the international health community, the PDA has primarily been used to study children who display exceptional growth despite living in impoverished environments (5). The PDA focuses on the strengths of a community, uses a problem-solving methodology, and empowers the community to lead the change. It is founded on a discovery over three decades ago that some individuals in a community who operate in a comparable socioeconomic context as their peers are achieving superior nutritional outcomes due to their special behaviors or strategies (6). Since the 1970s, maternal and child health programs have been employing this approach to tackle childhood malnutrition. Instead of fixating on what is not working, they focus on learning from and expanding upon what is already effective (7). Initially, PD was conceived in nutrition research and then put into practice to enhance nutrition outcomes in Vietnam. The triumph achieved in nutrition led to its subsequent implementation in more than 40 countries across the globe (8, 9).

Currently, positive deviance is being more utilized in international development activities as a way to incorporate locally tested solutions into problem-solving. It also serves as a method to encourage local engagement in addressing these issues (10). Individuals in the community who show exceptional behavior (uncommon behavior) enable them to achieve the best possible outcomes compared to their neighbors with the same resources (11, 12). ‘One approach to leveraging health-promoting parenting practices that persist in the face of stressful circumstances is by focusing on ‘Positive Deviance’ (13).

PD improved dietary intake, rapid weight gains in severely malnourished children, exclusive breastfeeding, and reduced morbidity in the intervention communities compared with non-intervention communities (14, 15). Positive results have been observed worldwide through the use of PDA. For instance, a study conducted in India utilized PDA to improve complementary feeding (16), in Mozambique and Burundi, PDA was applied to tackling undernutrition (10, 17), and in Ghana, household food security promotion and diet and growth trials (18) showed the application and contribution of PDA in different areas of public health (19).

In Ethiopia, there have been limited studies on the relationship between malnutrition and a positive deviance approach (PDA). PDA is a unique practice that provides an advantage to those who practice it compared with the rest of the community. This behavior is likely to be feasible, acceptable, and sustainable since it is already prevalent among at-risk populations, does not contradict local culture and traditions, and is effective. Therefore, this study aimed to assess the effectiveness of a community-based intervention using the PDA in enhancing mothers’ nutritional knowledge, attitudes, and self-efficacy and improving their children’s nutrition outcomes in rural Ethiopia.

## Materials and methods

### Study setting and period

This study was conducted in Maji Woreda, a rural setting, from April 15 to October 19, 2022. The details of the study setting have been described in a study published elsewhere (18).

### Study design and population

A parallel-group, single-blinded, cluster-randomized, two-arm trial was designed to investigate the effectiveness of a positive deviant approach (PDA) for improving mothers’ nutritional knowledge, attitude, and self-efficacy and children’s nutritional status. The trial followed the CONSORT recommendations for cluster randomized control trials (Campbell et al.). The intervention was conducted in an environment that promoted collective participation. Clusters were used as randomization units to prevent intervention contamination and improve logistical convenience. Clusters are the lowest administrative units, known as zones, in the kebele. Each kebele is divided into many small zones.

**Figure 1:**
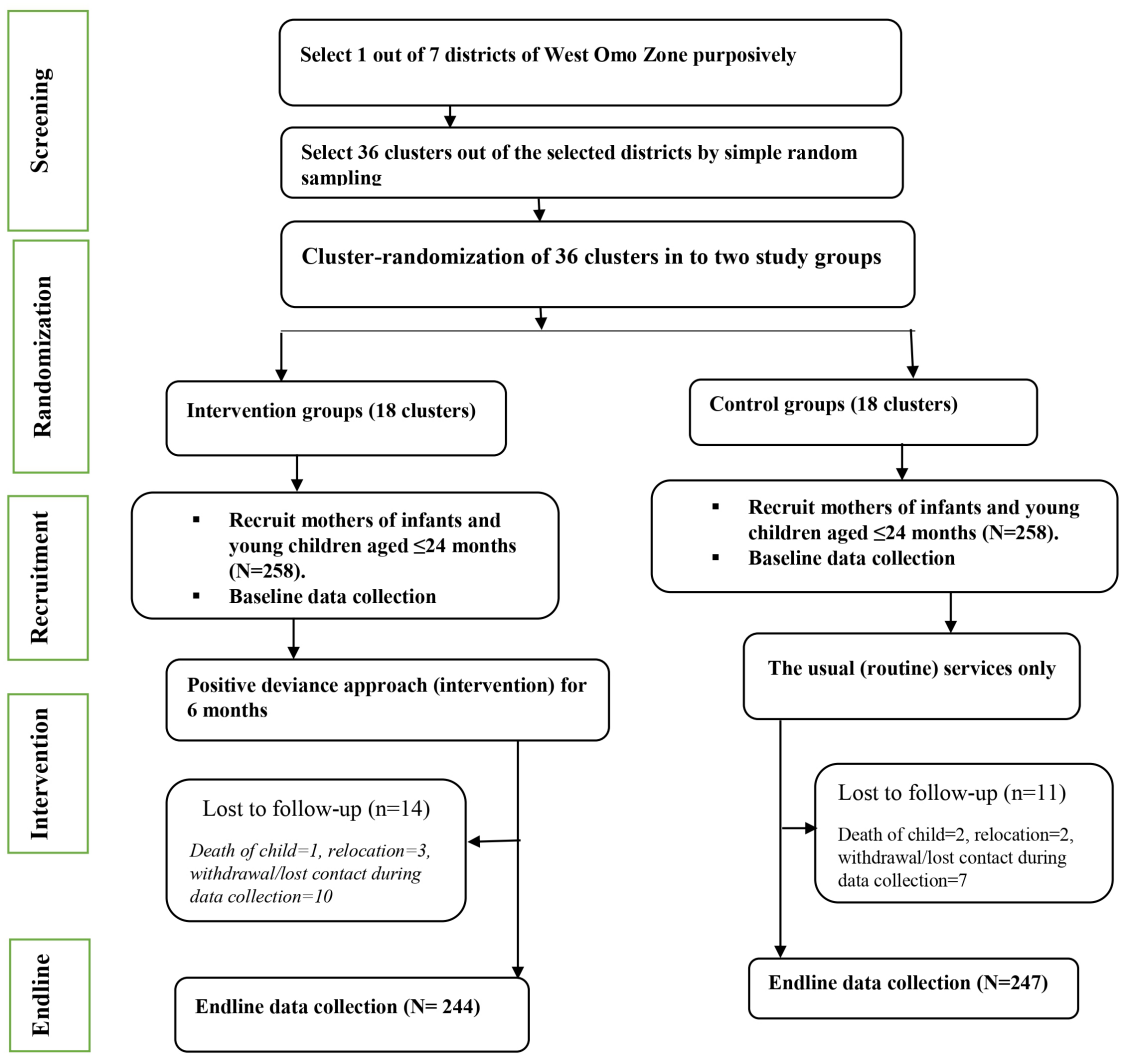
Trial profile.

Mothers who consented and had resided in the study area for at least 6 months before the study were enrolled. Participants who were unable to communicate due to illness were excluded (Figure 1). This study used a similar population as a study published elsewhere (20).

### Sample size determination

The sample size was calculated using statcalc (STATA software version 14) with the following assumptions: to detect an increase in appropriate feeding from 7 to 14% (3), with 95% CIs and 80% power, assuming an intra-class correlation coefficient of 0.03 (21). The total sample size was 516 mother–child pairs (258 from the intervention arm and 258 from the control arm). The details of the sample size determination have been described in a study published elsewhere (20).

### Sampling and randomization

We used a multistage sampling technique followed by a systematic random sampling technique to identify mothers with index infants and young children. In the first step, one woreda (district) was selected by simple random sampling (the lottery method). Second, lists of all kebeles (clusters) in the selected districts were compiled from the district administrative offices. A total of 36 non-adjacent clusters geographically accessible from 88 zones (small administrative units) were purposefully selected by listing them in alphabetical order, and a list of random numbers was generated in Microsoft Excel 2016 and fixed by being copied as “value” next to the alphabetical list of zones. According to the production, random numbers were placed in an ascending order. The last 18 zones were chosen as control clusters, and the first 18 served as intervention clusters. Third, 516 mothers were recruited using health extension workers’ family registration books to find mothers who had infants and children less than 24 months of age. An Excel sheet was formed from the logbook, and the households were selected using simple random sampling techniques.

Mothers within the zones served as the unit of observation, and zones in the kebeles served as the unit of randomization for the trials. We assigned the zones by simple randomization with a 1:1 allocation to either the control or intervention groups. The intervention assignment was concealed from the interviewers who collected the outcome data. Because of the nature of the intervention, mothers cannot be blind. All mothers, health extension workers, members of the women’s health army, and community volunteers were blind to the study’s hypothesis. The general objectives of the study were described in the agreement for data collection.

### Positive deviance approach

**Figure 2:**
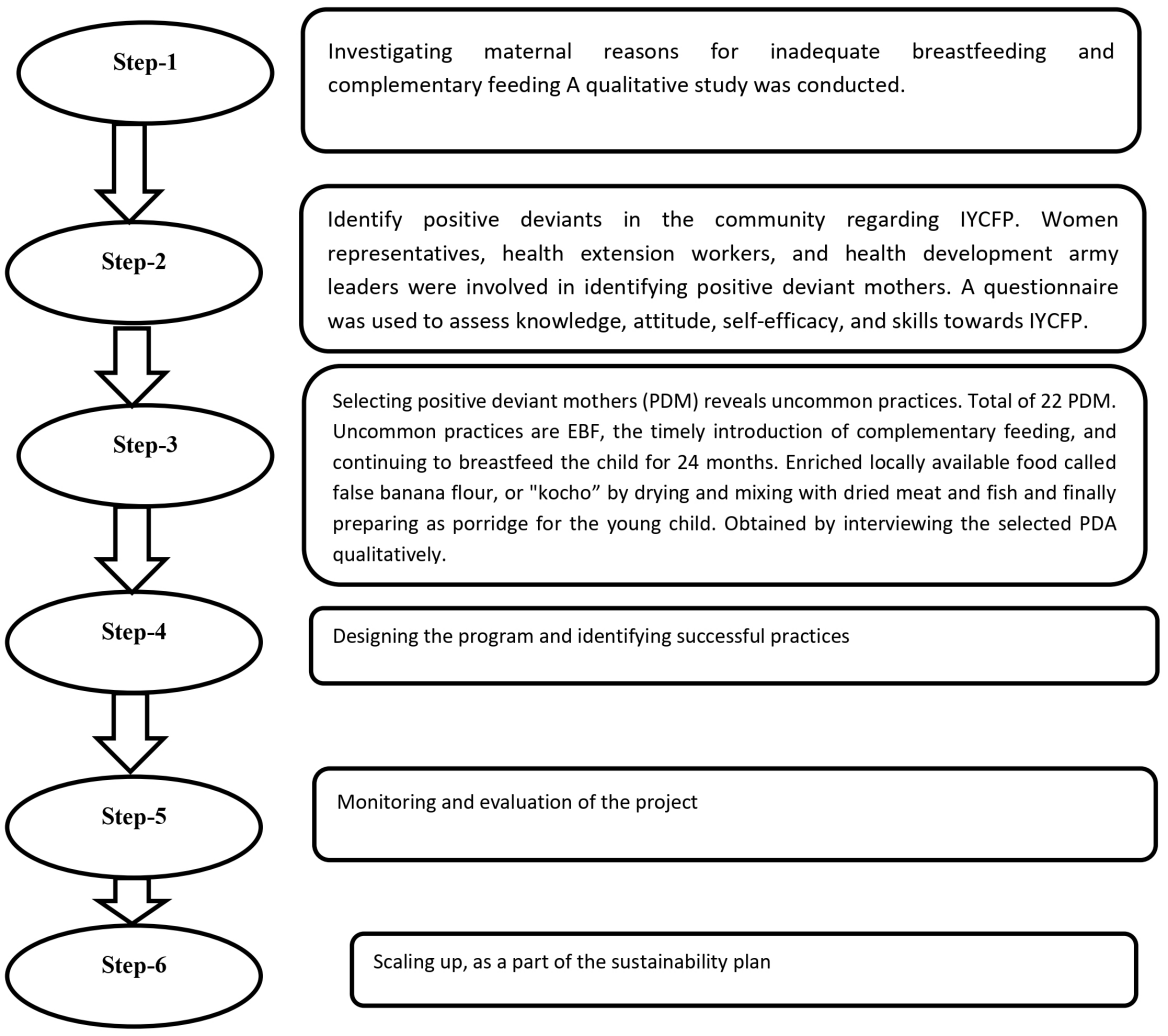
Positive deviant approach steps.

The researchers, village leaders, women representatives, health development army leaders (HDALs), and health extension workers (HEWs) collaborated on design artifacts, workflows, and work environments. Through repeated discussions, they were able to deepen and refine their understanding of the activities. This method allows researchers to comprehend the implicit and tacit knowledge that mothers possess but cannot express in words. This knowledge is holistic and extensive, and mothers possess a rich background of experiences that cannot be fully articulated. To better understand this knowledge, the team used positive deviant queries to identify and examine exemplary practices in specific settings. Six essential steps are carried out by a researcher, village leader, woman representative, HDAL, and HEW in the process (12). The details of the steps are presented in Figure 2.

### Intervention activities

#### Part 1: training of selected positive deviant mothers

Village leaders, women’s representatives, and health development army leaders suggested a total of 31 eligible PD mothers. Among them, 22 fulfill the requirement both at the mother’s and child’s sides. A two-week comprehensive training was provided to the mother based on the manual prepared for the training. The purpose of the training was to enrich the tacit knowledge they have about the recommended infant and young child feeding practices (IYCFP). PD plays a vital role in their communities by serving as counselors for infant and young child feeding (IYCF) and supporting group leaders. Equipped with practical knowledge, positive attitudes, and a strong sense of self-efficacy, these mothers effectively provide guidance, lead support groups, and engage in productive discussions with other mothers to encourage the adoption of the recommended IYCFP.

The key messages of the training session are as follows: optimal IYCFP, proper attachment of the breast to the child, and how to express and store breast milk. Regarding complementary feeding, we also conducted a cooking demonstration to teach people how to prepare and incorporate different types of locally accessible foods into homemade complementary meals. After the training, all PD mothers received a copy of the information education material prepared for the sessions, while the manual prepared for this training was given at the beginning of the training.

The instructional strategies delivered included direct, interactive, participatory, and experience-sharing activities. These strategies aimed to enhance knowledge, attitude, and self-efficacy through various methods such as talks, group discussions, experience sharing, group work exercises, demonstrations, role-play, storytelling, and problem-solving. The intervention’s key message focused on specific topics related to breastfeeding, such as the importance of early initiation and exclusive breastfeeding. It also addressed issues surrounding complementary feeding, including dietary diversity, minimum food frequency, and the appropriate time to start introducing complementary foods (*CF*). The intervention also emphasized the significance of enriching *CF* and provided step-by-step demonstrations on how to prepare enriched food items using locally available food options.

#### Part 2: group training of mothers with PDM

Nutrition education sessions, including demonstrations, were provided to selected mothers in the intervention group for 12 consecutive days in groups in their community setting for 90–120 min each day. The intervention consists of these elements: (a) providing education on breastfeeding to increase knowledge, attitude, and self-efficacy in breastfeeding; (b) offering support for complementary feeding; (c) counseling on methods to enhance consistency, quantity, and frequency of food intake using locally accessible food options; (d) conducting practical demonstrations on cooking locally available food items; (e) providing hygiene support; and (f) guiding parents on how to feed their child during and after an illness.

#### Part 3: home visits

Each positive deviant mother conducted a total of 12 home visits (twice a month) for the intervention group, aiming to bring the intended change to the maternal and child sides. During each home visit, counseling, sharing experiences of how they overcome their problems, and support were offered for the mother to reinforce the adoption of feeding practices she has been experiencing and to support them with training provided on optimum IYCFP. The mothers also demonstrated how to appropriately breastfeed and/or cook according to the age of the child. Moreover, feedback was given to the mothers participating in the trial. The participatory discussion was also carried out with the mother on the importance of breastfeeding and complementary feeding and their relevance for the health and growth of the child. Each mother was provided with a poster containing a key message for reinforcing the desired behavior.

Mothers were encouraged to inquire about any queries about the discussed topic. Additionally, a culturally suitable poster was used to visually depict information, such as the correct and incorrect methods of breastfeeding, the correct preparation of enriched flour, the appropriate thickness of complimentary food, as well as the inappropriate consistency. The poster also included images showcasing the advantages of adhering to the recommended IYCFP, presenting babies who were properly nourished versus those who were not.

All activities of PD mothers were supervised by the health professionals trained and recruited for supervision purposes. Overall supervision was also done by the researcher to get feedback from each positive deviant mother to identify if they had faced any challenges, such as technical as well as medical problems. The checklist was employed to verify the presence of positive deviant and non-deviant mothers according to the predetermined schedule. A sign was attached to it.

The feedback had been given, and a solution was sought for both technical and medical issues. If the medical issues were raised with both the mother and the child, immediate referrals to nearby health centers were made.

### Control group

The control groups received the routine health education provided by the health extension workers working in the kebele.

### Blinding

The data collectors were not aware of the allocation clusters, and they were also not residents in any of the clusters. However, the trial mothers were aware of the intervention allocation since the nature of the intervention made it evident, although its specific purpose was not disclosed.

### Process evaluation

A process evaluation was conducted to document the implementation process of the intervention and assess whether the intervention activities were carried out according to the plan. It also aimed to evaluate the performance of the PD mothers who provided the intervention and determine the extent to which the intervention reached the intended target mothers (22).

### Data collection methods and outcome measurements

Mothers who were enrolled in the study were surveyed at the beginning and end of the study using a structured questionnaire to gather information on child, maternal, and household characteristics. The mothers’ knowledge of breastfeeding was assessed based on 17 questions, with correct answers receiving a score of one and incorrect answers or unsure responses receiving a score of zero. The questionnaire used was adapted from a previous study among Chinese mothers, which was translated from English to Amharic (23). We decided to use cut-offs above and below the mean to dichotomize knowledge levels. Accordingly, all mothers who scored ≥ the mean in the knowledge test were considered to have a high level of knowledge, and those scoring below the mean were considered to have a low level of knowledge.

The mothers’ attitudes toward breastfeeding were evaluated using the Iowa Infant Feeding Attitude Scale, consisting of 17 items rated on a five-point Likert scale. Attitude toward breastfeeding was categorized as follows: (1) positive to breastfeeding (IIFAS score 70–85), (2) neutral (IIFAS score 49–69), and (3) positive to formula feeding (IIFAS score 17–48) (24). Similarly, the mothers’ self-efficacy toward breastfeeding was measured using the short form of the breastfeeding self-efficacy scale, which consisted of 14 items rated on a five-point Likert scale. All items are presented positively, and scores are summed to produce a range from 14 to 70. Breastfeeding self-efficacy was categorized as low self-efficacy (14–32 points), medium self-efficacy (33–51 points), and high self-efficacy (52–70 points).

Mothers’ knowledge, attitude, and self-efficacy about complementary feeding were assessed using a pretested questionnaire. The knowledge questionnaire consists of 10 items and consists of both open-ended and multiple-choice questions. Each question was scored 1 for correct and 0 for incorrect answers. The scores were summed, and a mean score for knowledge questions was computed. Respondents who scored less than the mean were labeled as having “low” knowledge, and those who scored equal to or above the mean were considered to have “high” knowledge.

Attitudes toward complementary feeding consisted of 8 items on a five-point Likert scale, rating maternal attitudes toward complementary feeding. A mean score for attitude questions was computed, and respondents who scored below the mean were considered to have an “unfavorable” attitude, and those who scored equal to or above the mean were considered to have a “favorable “attitude. Complementary feeding self-efficacy was measured by 9 items on a five-point Likert scale measuring maternal confidence. A mean score for self-efficacy questions was computed, and respondents who scored below the mean were considered to have “low” self-efficacy, while those who scored equal to or above the mean were considered to have “high “self-efficacy.

Infants and children’s length and weight were measured twice by two different teams of data collectors. The measurements were recorded on separate forms to ensure that the first measurement did not influence the second. Before each measurement, the weighing scale was calibrated to zero. Standard measures were obtained for height and weight (25, 26). For children aged 0–24 months, recumbent length was measured to the nearest 0.1 cm on a flat surface with a measuring tape and lying boards. For older children, standing height was measured to the nearest 0.1 cm, ensuring that the head, shoulder, buttocks, and heel all touched the vertical surface of the stadiometer.

### Data quality control

The questionnaire was originally developed in English and then translated into Amharic. It was then back-translated to English by an expert in the language to ensure consistency. To test the reliability of the questionnaire, a pretest was conducted on 5% of participants from the Bench-Sheko zone, which was outside of the study area. Cronbach’s alpha was calculated and found to be 0.88, which is within the acceptable range (>0.7), indicating good reliability. Before the actual data collection, supervisors and the researcher provided daily supervision and made necessary adjustments to the questionnaire.

### Data analysis

The intention-to-treat (ITT) principle was applied to all primary analyses. Double data entries were conducted using EpiData (version 3.1), and all statistical analyses were carried out using SPSS version 23. Descriptive statistics were used to present the baseline characteristics of the study groups. The chi-square test was utilized to assess baseline differences between the study groups for categorical variables, while the *t*-test was used for continuous variables. Generalized estimated equations (GEE) regression analyses, adjusted for clustering, were employed to examine the effect of the intervention on knowledge, attitude, and self-efficacy toward optimum young child feeding and nutritional outcomes in the intervention and control groups. The mean percentage difference in PDA mothers was assessed using a two-sample test of proportions. LAZ, WAZ, and WLZ indices were created based on the WHO child growth standards (WHO Multicentre Growth Reference Study Group), using length and weight measurements. Statistical significance was determined at a value of *p* of less than 0.05.

### Ethical approval and consent to participate

The ethical review committee of the Jimma University Institute of Health Research and Postgraduate Office (reference number: IHRPG/938/20) has approved a study. Written informed consent was obtained from the mother of every enrolled child. The study was conducted according to the guidelines in the Helsinki Declaration for research involving human participants.

## Results

Initially, a total of 516 pairs of mothers and young children (258 in the intervention group and 258 in the control group) were recruited, resulting in a 100% response rate. Fourteen (5.43%) in the intervention group had to be excluded due to reasons such as the death of a young child (*n* = 1), relocation from the study area (*n* = 3), voluntary withdrawal from the study, or lost contact during the collection of endline data (*n* = 10). Eleven (4.26%) in the control group had to be excluded due to reasons such as the death of a young child (*n* = 2), relocation from the study area (*n* = 2), voluntary withdrawal from the study, or lost contact during the collection of endline data (*n* = 7). The data collected at the endline phase involved 491 mothers (95.15%) who had actively participated in both groups.

### Baseline characteristics

Except for child sex and child age, baseline infant and young child, maternal, and household characteristics were comparable between the intervention and control groups. The details about the baseline characteristics are described in Table 1.

**Table 1:** Socio-demographic characteristics of mothers, in Maji District, West Omo Zone, Southwest Ethiopia.

Variables	Intervention (*n* = 258)		Control (*n* = 258)		*X*^2^ test	Value of *p*
	*n*	(%)	*n*	(%)		
Mother’s age (in years)						
18–24	38	14.23	38	14.23	0.07	0.967
25–34	130	51.55	136	51.55		
35–49	90	34.22	84	34.22		
**M ± SD**	31.69 ± 7.74		30.83 ± 7.01			
**Marital status**						
Married	247	95.74	251	97.30	0.92	0.337
Divorced	11	4.26	7	2.70		
**Religion**						
Orthodox Christian	166	64.34	171	66.28	0.21	0.644
Protestant	92	35.66	87	33.72		
**Maternal occupation**^**†**^						
Housewife/farmer	256	99.22	255	98.84		0.999
Government employee	2	0.78	3	1.16		
**Family size**						
1–3	58	22.48	58	22.48	3.02	0.221
4–6	131	50.77	147	56.98		
≥7	69	26.75	53	20.54		
**Monthly income of the household (ETB)**						
≤500	140	54.26	124	48.06	2.53	0.639
500–1,000	90	34.88	98	37.98		
1,000–1,500	14	5.43	16	6.20		
1,501–2000	8	3.10	12	4.65		
≥2000	6	2.33	8	3.11		
**Maternal educational status**						
Illiterate	97	37.58	91	35.27	0.30.	0.859
Primary school	141	54.65	146	56.59		
Secondary school and higher	20	7.77	21	8.14		
**Household food security status**						
Secured	14	5.43	13	5.04	0.04	0.843
Not secured	244	94.57	245	94.96		
**How long did you breastfeed your last child**						
<24 months	152	58.91	165	63.95	1.38	0.24
≥24 months	106	41.09	93	36.05		
**Child sex**						
Male	157	60.85	131	50.77	5.31	0.021*
Female	101	39.15	127	49.23		
**Child age (months)**						
0–5	41	15.89	45	17.44	15.03	0.002*
6–11	85	32.94	85	32.94		
12–17	118	45.74	90	34.88		
18–24	14	5.43	38	14.74		
**M ± SD**	10.97 ± 4.96		11.39 ± 5.80			
**Birth order**						
1st	45	17.44	44	17.05	0.09	0.958
2nd – 4th	171	66.28	174	67.44		
5th or more	42	16.28	40	15.51		
**Number of ANC visits**						
No ANC visits	118	45.74	116	44.96	0.04	0.981
<4 visits	112	43.41	113	43.8		
≥4 visits	28	10.85	29	11.24		
**Received breastfeeding information**						
No	174	67.44	179	69.38	0.22	0.636
Yes	84	32.56	79	30.62		
**Place of delivery**						
Home	173	67.05	184	71.32	1.1	0.294
Health institution	85	32.95	74	28.68		
**Delivery type**^**†**^						
Normal vaginal delivery	254	98.45	255	98.84		0.999
Caesarian section	4	1.55	3	1.16		
**First time breastfeeding**						
Immediately/within an hour of birth	91	35.27	98	37.98	0.87	0.833
After the first hour	130	50.39	127	49.22		
After 1 day	11	4.26	12	4.65		
Do not remember/ do not know	26	10.08	21	8.15		
**Postpartum complications**						
No	185	71.70	193	74.81	0.63	0.426
Yes	73	28.30	65	25.19		
**Postnatal care**						
No	214	82.94	225	87.21	1.85	0.174
Yes	44	17.06	33	12.79		
**Number of children**						
1–2	50	19.38	58	22.48	2.11	0.349
3–4	130	50.39	136	52.71		
≥5	78	30.23	64	24.81		
**Parity**						
Primiparous	45	17.44	44	17.05	0.01	0.907
Multiparous	213	82.56	214	82.95		

Chi^2^ test; *significant at *p* < 0.05; ^†^Fisher’s exact probability test.

### Effect of PDA on mothers’ breastfeeding knowledge, attitude, and self-efficacy

The results showed a mean difference of (MD) breastfeeding knowledge (MD = 6.47; 95% CI 6.45–6.49), breastfeeding attitude (MD = 12.68; 95% CI 11.96–13.40), and breastfeeding self-efficacy (MD = 3.13; 95% CI 3.05–3.21) favoring the intervention group. Furthermore, comparatively, the highest and lowest impact of the intervention (effect size) was reflected in breastfeeding attitude (ES = 34%) and breastfeeding self-efficacy (ES = 9%) (Table 2).

**Table 2:** Multivariate general linear modeling parameters for the effects of PDA on mother’s breastfeeding knowledge, attitude, and self-efficacy.

Outcomes	Mean (SD)			
	Intervention	Control	MD (95% CI)	Effect size
BFKQ score	13.28 (1.85)	6.81 (1.01)	6.47 (6.45 to 6.49)	0.29
IIFAS score	65.79 (5.98)	53.11 (5.33)	12.68 (11.96 to 13.40)	0.34
BSES-SF score	38.12 (3.33)	34.99 (2.99)	3.13 (3.05 to 3.21)	0.09

BFK, breastfeeding knowledge questionnaire; IIFAS, The Iowa infant feeding attitudes scale; BSES-SF, Breastfeeding Self-Efficacy Scale—Short Form; SD, standard deviation; MD, mean differences.

### Effect of PDA on mothers’ complementary feeding knowledge, attitude, and self-efficacy

Regarding PDA, the effect on the mother’s complementary feeding (CF) showed a mean difference in (MD) complementary feeding knowledge (MD = 4.53; 95% CI 4.31–4.75), *CF* attitude (MD = 9.14; 95% CI 8.52–9.75), and *CF* self-efficacy (MD = 11.64; 95% CI 11.16–12.12). Furthermore, comparatively, the highest and lowest impact of the intervention (effect size) was reflected in *CF* self-efficacy (ES = 31%) and *CF* knowledge (ES = 12%) (Table 3).

**Table 3:** Multivariate general linear modeling parameters for the effects of PDA on mother’s complementary feeding knowledge, attitude, and self-efficacy.

Outcomes	Mean (SD)			
	Intervention	Control	MD (95% CI)	Effect size
*CF* knowledge score	6.96 ± 1.09	2.43 ± 1.09	4.53 (4.31–4.75)	0.12
*CF* attitude score	28.55 ± 3.01	19.41 ± 2.75	9.14 (8.52–9.75)	0.29
*CF* self-efficacy score	32.52 ± 4.11	20.88 ± 3.85	11.64 (11.16–12.12)	0.31

CF, complementary feeding; SD, standard deviation; MD, mean differences.

### Effect of PDA on anthropometric outcomes

Positive deviant approach had no statistically significant effect on the prevalence of stunting and wasting in infants and young children in the intervention group compared to the control group (diff:6.46, 95% CI: 2.31, 11.97%; *p* = 0.21) and 9.03% (95% CI: 5.21, 14.03%; *p* = 0.121), respectively. The intervention group showed statistically significant differences and a greater decline in the prevalence of underweight (18.23%, 95% CI, 4.55, 22.54%; *p* = 0.004) compared with the control group (Table 4).

**Table 4:** Effect of PDA on anthropometric outcomes at 6 months of follow-up.

Outcomes	Control area		Intervention area		Absolute percentage (%) difference 95% CI ^†^	Value of *p*
	n*	*n* (%)	n*	*n* (%)		
Stunting (LAZ < -2)					−6.46(−11.97,-2.31)	0.21
Baseline	258	70 (27.13)	258	78 (30.23)		
Endline	247	65 (26.31)	244	60 (24.59)		
**Underweight (WAZ < -2)**					−18.23 (−22.54,-4.55)	0.004
Baseline	256	61 (23.83)	256	70 (27.34)		
Endline	245	53 (21.63)	243	27 (11.11)		
**Wasting (WLZ < -2)**						
Baseline	256	33 (12.89)	256	36 (14.06)	−9.03(−13.04,-5.21)	0.121
Endline	245	29 (11.84)	243	15 (6.17)		

*At baseline, the total number of children surveyed was 516 (*n* = 258 in the intervention area and *n* = 258 in control areas), with no weight measurement (*n* = 7) and treated as missing values through. ^†^Estimated by mixed effect linear regression analysis, adjusting for enrollment status in growth outcomes, child age and sex. Length-for-age (LAZ), weight-for-age (WAZ), weight-for-length (WLZ).

## Discussion

### Impact of the intervention

This study utilized cluster randomized control trials to demonstrate the effectiveness of the Positive Deviant Approach (PDA) in enhancing various outcomes related to breastfeeding, complementary feeding, and child nutritional status among rural Ethiopian mothers and children. Specifically, the use of PDA resulted in increased knowledge, improved attitudes, and greater self-efficacy toward breastfeeding and complementary feeding. Moreover, the study indicated notable improvements in weight-for-age *z*-scores (WAZ) and a reduction in cases of underweight among the children. The findings from this study are of utmost importance to public health as they depict the effectiveness of PDA in significantly improving child nutrition among rural Ethiopian mothers and children. Furthermore, the notable improvements in WAZ and a reduction in cases of underweight among children indicate the positive impact of PDA on child nutrition. These findings highlight the potential of implementing PDA as a strategy to address malnutrition and improve the overall health and well-being of rural communities.

The role of knowledge in health behavior change is widely acknowledged. The study discovered that the intervention group demonstrated increased scores in breastfeeding knowledge when compared to the control group. This study aligns with previous research conducted in Ethiopia (27) and India (28), which also found that PDA effectively increased breastfeeding knowledge scores in the intervention group compared to the control group. However, this study contradicts a breastfeeding education and support intervention conducted in Ethiopia, where no notable difference was observed between the intervention and control groups in terms of breastfeeding knowledge scores (29). These differences may be explained by the nature of PDA, where the community’s tacit knowledge was supported scientifically to solve its problems.

The current study showed the effect of PDA on improving breastfeeding attitudes in the intervention group compared to the control group at the endline. This study is in congruence with previous studies done in Ethiopia using PDA (27) and breastfeeding education and support interventions (29). The repeated exposure and positive, enduring evaluation of the importance of breastfeeding led the mother to develop a positive attitude toward breastfeeding, which contributed to an improved breastfeeding attitude score.

Self-efficacy significantly contributes to behavior change efforts. Higher levels of self-efficacy lead to increased motivation, perseverance, and confidence in one’s abilities, which can ultimately result in sustained behavior change. This study showed PDA improved mothers’ breastfeeding self-efficacy in the intervention group compared to the control group. This finding is in line with previous studies done in Ethiopia (27) and Malaysia (30).

This study revealed that PDA affects mothers’ complementary feeding (*CF*) knowledge in the intervention group when compared with the control group. Our finding is in line with the study done in Ghana (31). The PDA also had an effect on the *CF* attitude score in the intervention group compared with the control group. This is because PDA may have changed the mothers’ attitudes toward complementary feeding. The mothers previously assumed that complementary feeding was not affordable in their setting, and this intervention showed them how to use locally available food items for feeding their young children. This study’s finding is in line with a community-based nutritional education intervention conducted in Kenya (32).

In our study, PDA improved complementary feeding self-efficacy among mothers in the intervention group compared with the control group. It has been demonstrated that self-efficacy is a powerful motivator for positive health behavior changes (33). In this study, the mothers in the intervention groups were provided with information and skills that potentially enhanced their confidence in providing complementary feeding for their young children. Notably, the results of this study align with those of a positive deviant intervention study conducted in Ecuador (34), which witnessed an improvement in complementary feeding self-efficacy among the mothers in the intervention group compared to the control group.

In this study, PDA had no statistically significant effect on the prevalence of stunting in infants and young children in the intervention group compared to the control group. The findings of this study are in congruence with trials conducted in Malawi (35) and Cambodia (36), which used nutrition education interventions. The finding of this study is contrary to the study done in Ethiopia, which used PDA (37), where the intervention had a significant effect on stunting among the intervention group. This difference can be explained by the fact that in our study, the intervention was for 6 months and in the previous study, the intervention was for 12 months, which may contribute to the change.

In addition, this study revealed that PDA showed a statistically significant effect on the declining prevalence of underweight children compared with the control group. The results of this study are consistent with those of similar experiments conducted in Ecuador and Cambodia (34, 36), which also used positive deviant/health nutrition and showed that at follow-up, children in the intervention arm had improvements in weight-for-age and the likelihood of being underweight was reduced for children in the intervention arm. This study also revealed that PDA has no statistically significant effect on wasting in the intervention group when compared with the control group. The findings of this study are in line with trials conducted in Ethiopia that used PDA (37).

### Implication of the study

This intervention study is quite relevant and contributes to the field of public health and particularly to nutrition programs by inspiring the public health practitioner toward community-based participatory approaches to resolving nutrition-related issues in the study area and beyond. The study showed the effectiveness of PDA in improving maternal nutrition-related knowledge, attitude, self-efficacy, and a child’s nutritional status (reducing underweight) in the study area. This study also encourages future researchers to solve community problems with solutions that emerge from the community.

### Strengths and limitations of the study

The strength of our study was the utilization of a cluster randomized study design, which encompassed a relatively large sample size. Additionally, we employed a statistical approach to account for correlations within individual children and clusters. The inclusion of positive deviant mothers from the community further encouraged the sustainability of the intervention within the community. We ensured the use of validated tools to collect data on knowledge, attitude, and self-efficacy concerning both breastfeeding and complementary feeding. However, this study does have some limitations. Firstly, this study may introduce a social desirability bias into certain outcomes. Secondly, our study only involved two data collection sessions, conducted at baseline and endline. As a result, we recommend future studies include additional follow-up visits with enhanced enabling factors in place. This will aid in accurately capturing changes in mothers’ knowledge, attitude, self-efficacy, and nutritional outcomes.

## Conclusion

In conclusion, this research demonstrates that PDA can improve a mother’s nutritional knowledge, attitude, self-efficacy, and child’s nutritional status (WAZ). Hence, incorporating PDA with government nutrition programs in rural communities can potentially enhance mothers’ knowledge, attitude, self-efficacy, and nutritional outcomes. It is important to address the mother’s nutritional knowledge, attitude, and self-efficacy in promoting actual behavior. Therefore, these findings can inspire community-based participatory approaches to nutritional interventions. Additionally, there is a need to conduct further research to comprehensively understand the mechanisms by which PDA affects feeding practices.

## Data availability statement

The original contributions presented in the study are included in the article/supplementary material, further inquiries can be directed to the corresponding author.

## Ethics statement

The studies involving humans were approved by Jimma University’s Institute of Health Research and Postgraduate Office, Institutional Review Board. The studies were conducted in accordance with the local legislation and institutional requirements. The participants provided their written informed consent to participate in this study.

## Author contributions

AG: Conceptualization, Data curation, Formal analysis, Investigation, Methodology, Software, Validation, Writing – original draft, Visualization. PS: Conceptualization, Investigation, Methodology, Supervision, Validation, Visualization, Writing – review & editing, Software. MS: Conceptualization, Methodology, Supervision, Validation, Visualization, Writing – original draft, Writing – review & editing.

## References

[1] PAHO/WHO. Guiding principles for complementary feeding of the breastfed child. Washington, DC: Pan American Health Organization/ World Health Organization (2015).

[2] WHO/UNICEF. Infant and young child feeding counseling: an integrated course. World Health Organization. (2016)

[3] Central Statistical Agency (CSA) [Ethiopia] and ICF. Ethiopia demographic and health survey 2016. Rockville, Maryland, USA: Addis Ababa, Ethiopia: Central statistical agency [Ethiopia] and ICF. Ministry of Health Ethiopia. (2016)

[4] Vazir S. Behavioral aspects of development of eating behavior and nutrition status. Nutr Rev. (2002) 60:S95–S101. doi: 10.1301/00296640260130821 12035868

[5] Zeitlin M. Nutritional resilience in a hostile environment: positive deviance in child nutrition. Nutr Rev. (1991) 49:259–68. doi: 10.1111/j.1753-4887.1991.tb07417.x, PMID: 1749535

[6] Baik D Reinsma K Chhorvann C Oy S Heang H Young MF. Program impact pathway of the positive deviance/hearth interactive voice calling program in a Peri-urban context of Cambodia. Curr Develop Nutrition. (2022) 6:nzac045. doi: 10.1093/cdn/nzac045 PMC9121803 35611354

[7] Schooley J Morales L. Learning from the community to improve maternal–child health and nutrition: the positive deviance/hearth approach. J Midwifery Womens Health. (2007) 52:376–83. doi: 10.1016/j.jmwh.2007.03.001, PMID: 17603960

[8] Ahrari M Houser RF Yassin S Mogheez M Hussaini Y Crump P. A positive deviance-based antenatal nutrition project improves birth-weight in upper Egypt. J Health Popul Nutr. (2006) 24:498–507. PMID: 17591347 PMC3001154

[9] Ndiaye M Siekmans K Haddad S Receveur O. Impact of a positive deviance approach to improve the effectiveness of an iron-supplementation program to control nutritional anemia among rural Senegalese pregnant women. Food Nutr Bull. (2009) 30:128–36. doi: 10.1177/156482650903000204 19689091

[10] Levinson FJ Barney J Bassett L Schultink W. Utilization of positive deviance analysis in evaluating community-based nutrition programs: an application to the Dular program in Bihar. India Food Nutr Bull. (2007) 28:259–65. doi: 10.1177/156482650702800301, PMID: 17974358

[11] Fowles ER Hendricks JA Walker LO. Identifying healthy eating strategies in low-income pregnant women: applying a positive deviance model. Health Care Women Int. (2005) 26:807–20. doi: 10.1080/07399330500230953, PMID: 16214795

[12] Sternin M Sternin J Marsh D. Designing a community-based nutrition program using the hearth model and the positive deviance approach: A field guide. Westport, CT: Save the Children Federation (1998).

[13] Pascale RT Sternin J Sternin M. The power of positive deviance: How unlikely innovators solve the world's toughest problems Harvard Business School Pr. (2016)

[14] Doskey S Mazzuchi T Sarkani S. Positive deviance approach for identifying next-generation system engineering best practices. Procedia Comput Sci. (2013) 16:1112–21. doi: 10.1016/j.procs.2013.01.117

[15] Barbosa CE Masho SW Carlyle KE Mosavel M. Factors distinguishing positive deviance among low-income African American women: a qualitative study on infant feeding. J Hum Lact. (2017) 33:368–78. doi: 10.1177/0890334416673048, PMID: 27881731

[16] D'alimonte MR. Qualitative exploration of behaviors related to positive child growth in an urban slum of Mumbai. University of Yale (2014). Available at: elischolar.library.yale.edu

[17] Sosanya ME Adeosun FF Okafor DT Ifitezue LC. Positive deviance—an expeditious tool for action to ameliorate malnutrition in resource-poor settings. J Nutr Ecol Food Res. (2017) 4:178–87. doi: 10.1166/jnef.2017.1165

[18] Saaka M Mutaru S. Promoting household food and nutrition security in northern Ghana, vol. 46. Food Science & Nutrition. (2017)

[19] Dearing JW Singhal A. New directions for diffusion of innovations research: dissemination, implementation, and positive deviance. Hum Behav Emerg Technol. (2020) 2:307–13. doi: 10.1002/hbe2.216

[20] Gizaw AT Sopory P Morankar S. Breastfeeding knowledge, attitude, and self-efficacy among mothers with infant and young child in rural Ethiopia. PLoS One. (2022) 17:e0279941. doi: 10.1371/journal.pone.0279941, PMID: 36584131 PMC9803198

[21] Moss C Bekele TH Salasibew MM Sturgess J Ayana G Kuche D. Sustainable undernutrition reduction in Ethiopia (SURE) evaluation study: a protocol to evaluate impact, process and context of a large-scale integrated health and agriculture programme to improve complementary feeding in Ethiopia. BMJ Open. (2018) 8:e022028. doi: 10.1136/bmjopen-2018-022028, PMID: 30030320 PMC6059290

[22] Abiyu C Belachew T. Effect of complementary feeding behavior change communication delivered through community-level actors on dietary adequacy of infants in rural communities of west Gojjam zone, Northwest Ethiopia: a cluster-randomized controlled trial. PLoS One. (2020) 15:e0238355. doi: 10.1371/journal.pone.0238355, PMID: 32881945 PMC7470293

[23] Vijayalakshmi P Susheela DM. Knowledge, attitudes and breast feeding practices of postnatal mothers: a cross sectional survey. Int J Health Sci (Qassim). (2015) 9:363–72. doi: 10.12816/0031226 PMC4682591 26715916

[24] Chen S Binns CW Liu Y Maycock B Zhao Y Tang L. Attitudes towards breastfeeding—the Iowa infant feeding attitude scale in Chinese mothers living in China and Australia. Asia Pac J Clin Nutr. (2013) 22:266–9. doi: 10.6133/apjcn.2013.22.2.09, PMID: 23635372

[25] World Health Organization. WHO child growth standards: growth velocity based on weight, length and head circumference: methods and development, vol. 2 Geneva, Switzerland: World Health Organization. (2009)

[26] Yonas F. Infant and young child feeding practice status and associated factors among mothers of under 24-month-old children in Shashemene Woreda, Oromia region, Ethiopia. Open Access Library J. (2015) 02:1–15. doi: 10.4236/oalib.1101635

[27] Siraneh Y Woldie M Birhanu Z. Effectiveness of positive deviance approach to promote exclusive breastfeeding practice: a cluster randomized controlled trial. Risk Manage Healthcare Policy. (2021) 14:3483–503. doi: 10.2147/RMHP.S324762 PMC8403074 34466041

[28] Srivastava A Gwande K Bhattacharya S Singh VK. Impact of the positive deviance approach on breastfeeding practices among tribal pregnant women: a before–after intervention study. CHRISMED J Health Res. (2019) 6:222. doi: 10.4103/cjhr.cjhr_165_18

[29] Abdulahi M Fretheim A Argaw A Magnus JH. Breastfeeding education and support to improve early initiation and exclusive breastfeeding practices and infant growth: a cluster randomized controlled trial from a rural Ethiopian setting. Nutrients. (2021) 13:1204. doi: 10.3390/nu13041204, PMID: 33917366 PMC8067429

[30] Pilus FM Ahmad N Zulkefli NA Shukri NH. Effect of face-to-face and WhatsApp communication of a theory-based health education intervention on breastfeeding self-efficacy (SeBF intervention): cluster randomized controlled field trial. JMIR Mhealth Uhealth. (2022) 10:e31996. doi: 10.2196/31996, PMID: 36103244 PMC9520384

[31] Saaka M Wemah K Kizito F Hoeschle-Zeledon I. Effect of nutrition behaviour change communication delivered through radio on mothers’ nutritional knowledge, child feeding practices and growth. J Nutr Sci. (2021) 10:e44. doi: 10.1017/jns.2021.35, PMID: 34164123 PMC8190717

[32] Hitachi M Wanjihia V Nyandieka L Francesca C Wekesa N Changoma J. Improvement of dietary diversity and attitude toward recommended feeding through novel community based nutritional education program in coastal Kenya—an intervention study. Int J Environ Res Public Health. (2020) 17:7269. doi: 10.3390/ijerph17197269, PMID: 33027966 PMC7579186

[33] Bandura A. Health promotion from the perspective of social cognitive theory. Psychol Health. (1998) 13:623–49. doi: 10.1080/08870449808407422

[34] Roche ML Marquis GS Gyorkos TW Blouin B Sarsoza J Kuhnlein HV. A community-based positive deviance/hearth infant and young child nutrition intervention in Ecuador improved diet and reduced underweight. J Nutr Educ Behav. (2017) 49:196–203.e1. doi: 10.1016/j.jneb.2016.10.007 27843127

[35] Kuchenbecker J Reinbott A Mtimuni B Krawinkel MB Jordan I. Nutrition education improves dietary diversity of children 6-23 months at community-level: results from a cluster randomized controlled trial in Malawi. PLoS One. (2017) 12:e0175216. doi: 10.1371/journal.pone.0175216, PMID: 28426678 PMC5398527

[36] Young MF Baik D Reinsma K Gosdin L Rogers HP Oy S. Evaluation of mobile phone-based positive deviance/hearth child undernutrition program in Cambodia. Matern Child Nutr. (2021) 17:e13224. doi: 10.1111/mcn.13224, PMID: 34414653 PMC8476410

[37] Kang Y Kim S Sinamo S Christian P. Effectiveness of a community-based nutrition programme to improve child growth in rural Ethiopia: a cluster randomized trial. Matern Child Nutr. (2017) 13:1–15. doi: 10.1111/mcn.12349, PMID: 27549570 PMC6866137

